# The Role of Deubiquitinases in Oncovirus and Host Interactions

**DOI:** 10.1155/2019/2128410

**Published:** 2019-07-18

**Authors:** Yueshuo Li, Feng Shi, Jianmin Hu, Longlong Xie, Ann M. Bode, Ya Cao

**Affiliations:** ^1^Key Laboratory of Carcinogenesis and Invasion, Chinese Ministry of Education, Department of Otolaryngology Head and Neck Surgery, Xiangya Hospital, Central South University, Changsha 410078, China; ^2^Cancer Research Institute and School of Basic Medical Science, Xiangya School of Medicine, Central South University, Changsha 410078, China; ^3^Key Laboratory of Carcinogenesis, Chinese Ministry of Health, Changsha 410078, China; ^4^The Hormel Institute, University of Minnesota, Austin, MN 55912, USA; ^5^Research Center for Technologies of Nucleic Acid-Based Diagnostics and Therapeutics Hunan Province, Changsha 410078, China; ^6^Molecular Imaging Research Center of Central South University, Changsha 410008, Hunan, China; ^7^National Joint Engineering Research Center for Genetic Diagnostics of Infectious Diseases and Cancer, Changsha 410078, China

## Abstract

Infection-related cancer comprises one-sixth of the global cancer burden. Oncoviruses can directly or indirectly contribute to tumorigenesis. Ubiquitination is a dynamic and reversible posttranslational modification that participates in almost all cellular processes. Hijacking of the ubiquitin system by viruses continues to emerge as a central theme around the viral life cycle. Deubiquitinating enzymes (DUBs) maintain ubiquitin homeostasis by removing ubiquitin modifications from target proteins, thereby altering protein function, stability, and signaling pathways, as well as acting as key mediators between the virus and its host. In this review, we focus on the multiple functions of DUBs in RIG-I-like receptors (RLRs) and stimulator of interferon genes (STING)-mediated antiviral signaling pathways, oncoviruses regulation of NF-*κ*B activation, oncoviral life cycle, and the potential of DUB inhibitors as therapeutic strategies.

## 1. Introduction

About 15-16% of cancer cases are attributable to infection [1]. Viral infection is one of the main risk factors for the development of infection-related cancers. Currently, the known oncogenic viruses include Epstein-Barr virus (EBV) [2–4], Kaposi sarcoma herpes virus (KSHV) [5], human T-cell lymphotropic virus type 1 (HTLV-1) [1, 6], hepatitis B virus (HBV), hepatitis C virus (HCV), human papillomavirus (HPV), and human immunodeficiency virus type 1 (HIV-1) [7]. EBV, also known as human herpes virus 4, was the first virus to be associated with human malignancy. EBV is a double-stranded DNA virus. EBV infects approximately 95% of the world's population, which is the most common and persistent viral infection in humans. HTLV was the first human retrovirus to be identified. About 3–5% of HTLV-1-infected individuals develop adult T-cell leukemia/lymphoma (ATL), which is an aggressive and lethal malignancy with few effective therapeutic options [8]. Hepatocellular cancer (HCC) is the fifth most prevalent malignant tumor and the third leading cause of cancer-related deaths. HCC is a highly lethal cancer and is mainly associated with chronic HBV and HCV infections with about 80% of HCC caused by HBV and HCV infections [9]. Around 5% of global human cancers are caused by HPV [10]. HIV infection increases cancer risk mostly by immunosuppression and chronic immune activation [7] (Table 1).

The fate and function of most proteins depend on posttranslational modifications [11]. Ubiquitin is a posttranslational modifier and a key regulatory molecule participating in various cellular activities. Aberrant ubiquitin system activity is linked to many diseases, including cancer [12], infection [13, 14], and neurodegeneration [15]. All viruses need host machinery to maintain infection and replication. Therefore, oncoviruses rely on the ubiquitin system at many levels, and even hijack the ubiquitin system to satisfy their survival needs. Ubiquitination is dynamic and it can be reversed by deubiquitinating enzymes (deubiquitinases or DUBs). This explains why DUBs are the main regulators in the interactions between the virus and its host. Some viruses even encode viral deubiquitinating enzymes to affect multiple host cell processes. However, relevant research findings are very limited. Thus far, identifying and taking full advantage of viral-related DUBs is a continuing challenge [13]. Here, we review current knowledge from both the host and viral points of view, discussing how the DUBs are involved in the viral life cycle and how oncoviruses avoid or utilize the DUBs to satisfy their survival needs.

## 2. General Functions of DUBs

DUBs maintain ubiquitin system homeostasis by cleaving polyubiquitin chains or completely removing ubiquitin chains from ubiquitinated proteins and then generating and recycling free ubiquitin [16]. Deubiquitination has important functions in regulating the ubiquitin-dependent pathways, including cell cycle regulation, cell death, protein degradation, protein function, gene expression, and signal transduction [17]. Thus far, about 100 DUBs have been identified in six different families and are classified into two categories (Table 2) [18, 19]. Imbalances in DUBs activities are involved in multiple diseases, including cancer, inflammation, neurological disorders, and microbial infections [17]. DUBs, such as A20, OTULIN, and CYLD, mediate NF-*κ*B and cell death to maintain optimal signal transduction and immune homeostasis [20]. Compared with normal cells, cancer cells need elevated synthesis of growth-promoting proteins and protein-degradation capacity to satisfy uncontrolled mitosis. Much research has focused on studying their function and substrates to elucidate the role of DUBs in specific diseases. Abnormal expression of DUBs-encoding genes has been detected in human cancers. A mutant tumor suppressor gene* CYLD* has been identified in familial cylindromatosis and CYLD is downregulated in multiple cancer types [21]. Hajek,* et. al *have identified a distinct subset of HPV-associated head and neck squamous cell carcinomas that have* TRAF3/CYLD *mutations [22]. Multiple oncoviruses utilize these DUBs to edit ubiquitin chains and alter ubiquitin signaling, contributing to virus infection, replication, and pathogenesis. To date, vaccines against HBV and HPV have already begun to decrease the incidence of cancers attributed to these oncoviruses. However, other oncoviruses have no existing vaccines. In addition to prevention by vaccines, targeting the interplay between oncoviruses and their host might give rise to effective and inexpensive treatment strategies with minimal toxicity.

## 3. DUBs Participate in Antiviral Innate Responses

As the first line of host defense against viral infection, host pattern recognition receptors (PRRs), including RLRs, toll-like receptors (TLRs), and cytosolic dsDNA sensors (such as STING), recognize viral nucleic acids inducing innate immune responses, resulting in the production of type I interferons (IFNs) and proinflammatory cytokines [23, 24]. Using or bypassing host immune signaling is important for viruses to successfully establish infection. A thorough understanding of the molecular mechanisms between virus-related deubiquitination and antiviral innate immunity signaling is necessary for the control of infectious diseases and for developing therapeutic targets.

### 3.1. DUBs Are Involved in RLRs-Mediated Innate Immunity against RNA Oncoviruses

**Figure 1:**
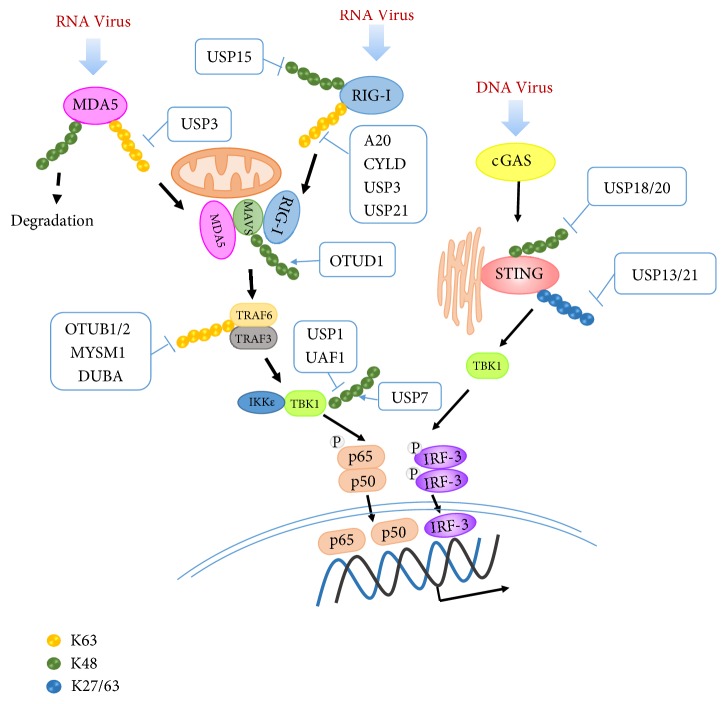
*DUBs participate in antiviral innate immunity. *During virus infection, K63-linked polyubiquitination of RLRs promotes their interaction with MAVS and signal transmission. USP15 inhibits K48-ubiquitination of RNA sensor RIG-I to inhibit RIG-I degradation; A20, CYLD, USP3, and USP21 inhibit K63-ubiquitination of RIG-I to negatively regulate RIG-I activation. USP3 inhibits K63 ubiquitination of MDA5 to inhibit its activation. RIG-I and MDA5 bind to and activate MAVS. Activated MAVS works as a scaffold to recruit various TRAFs, leading to TBK1/I*Ƙ*B kinase *Ɛ* (IKK-*Ɛ*)-mediated phosphorylation and nuclear translocation of IRF3 and IRF7, and production of IFNs and OTUD1 stabilizes MAVS by removing K48-ubiquitination. Deubiquitinases OTUB1/2, MYSM1, and DUBA inhibit K63-linked ubiquitination of TRAF3 or TRAF6 and negatively regulate IFNs production. HSV infection can recruit USP21 to deubiquitinate the K27/63-linked polyubiquitin chain on STING. USP13 removes K27-linked polyubiquitin chains from STING and thereby impairs the recruitment of TBK1 to reduce the antiviral immune response against DNA viruses. USP18 recruits USP20 in an enzymatic activity-independent manner and facilitates USP20 to remove K33- and K48-linked ubiquitin chains from STING, thereby preventing degradation of STING caused by DNA virus infection. USP7 interacts with TRIM27 and removes its K48-linked polyubiquitination, promoting the degradation of TBK1. USP1 and UAF1 inhibit K48 polyubiquitin chains to stabilize TBK1 contributing to IFNs production.

RNA viruses are mainly recognized by RLRs. RLRs recognize viral RNAs through the RNA helicase domain (RLD), and then interact with the mitochondrial antiviral signaling protein, MAVS [25]. The RLRs include retinoic acid-inducible gene I (RIG-I) and melanoma differentiation-associated gene 5 (MDA5), which belong to a family of cytosolic host RNA helicases that recognize distinct nonself RNA signatures and trigger innate immune responses against several RNA viral infections. After recognition of viral RNA through the RNA helicase domain (RLD), RIG-I or MDA5 binds to MAVS. The K63-linked polyubiquitination of these adaptors is essential for signal activation. On the other hand, DUBs have also been shown to regulate antiviral innate immunity. Some DUBs negatively regulate the innate immune system to guard against excessive self-destructive immune responses and thus play a critical role in maintaining the balance of the immune system. USP21 [26], USP3 [27], and CYLD [28] negative regulate RIG-I and MDA5 activation by binding to and removing K63-linked polyubiquitin chains. The deubiquitinases OTUB1/2 [29, 30] and MYSM1 [31] inhibit K63-linked ubiquitination of TRAF3/6 and negatively regulate IFNs production. OTUD1 can also remove K48-linked ubiquitination from Smurf1, which targets MAVS for K48-linked ubiquitination and degradation, contributing to the degradation of MAVS [25]. Zhang* et al.* found that RNA viral infection can utilize the OTUD1-Smurf1 axis through the NF-*κ*B signaling pathway to promote downregulation of the MAVS, TRAF3, and TRAF6 proteins and IFNs production [32]. In addition to the DUBs mentioned above, the host also uses positive regulation of DUBs against viral infection. USP15 reduces the K48-linked ubiquitination of TRIM25 (targeting RIG-I K63-linked ubiquitination and activation) leading to its stabilization [33] and promoting RIG-I activation. USP25 clears virus-triggered K48-linked ubiquitination, promoting the stability of TRAF3 and TRAF6 [34] and positively regulating RNA virus-triggered innate immune responses. USP1 and UAF1 bind to TBK1, remove its K48-linked polyubiquitination, and reverses the degradation process of TBK1. This USP1–UAF1 complex enhances TLR3/4 and RIG-I–induced IFN regulatory factor 3 (IRF3) activation and subsequent IFN-*β* secretion [35]. These studies indicate that DUBs play a critical role in regulating the virus-triggered RIG-I-like pathway and IFNs production, which are crucial for RNA viruses to establish efficient infection at an early stage (Figure 1).

### 3.2. DUBs Are Involved in STING-Mediated Innate Immunity against DNA Oncoviruses

Host cells express multiple cytosolic DNA sensors to recognize exogenous viral nucleic acids, such as DAI, DDX41, IFI16, and cyclic GMP-AMP synthase (cGAS). These sensors trigger signaling pathways and activate the adaptor protein stimulator of IFN genes (STING; also known as MITA) to induce the expression of type I IFN [36]. STING is a key adaptor protein for most DNA sensing pathways. Ubiquitination of STING caused by viral infection plays critical roles in virus-triggered signaling [37]. K27- or K63-linked ubiquitination mediated by various E3 ubiquitin ligases, such as TRIM32, AMFR, and INSIG1 [38, 39], is essential for full activation of STING. Double-stranded DNA viruses, such as EBV, use ubiquinase TRIM29 to ubiquitinate and degrade STING, suppressing host innate immunity that leads to the persistence of DNA viral infections [40]. HSV infection can recruit USP21 to STING through p38-mediated phosphorylation of USP21 at Ser538. USP21 deubiquitinates the K27/63-linked polyubiquitin chain on STING, thereby leading to reduced production of type I IFNs [41]. During HTLV-1 and HBV infection, Tax and HBV polymerases decrease the K63-linked ubiquitination of STING and disrupt the interactions between STING and TBK1, which leads to loss of STING function and subsequent impairment of IRF3 activation, IFN-induction, and an antiviral response [42, 43]. In addition, USP13 removes K27-linked polyubiquitin chains from STING and then decreases the antiviral immune response against DNA viruses by disrupting the recruitment of TBK1 [44]. To inhibit DNA viral infection, USP18 recruits USP20 in an enzymatic activity-independent manner and facilitates USP20 to remove K33- and K48-linked ubiquitin chains from STING, thereby preventing degradation of STING caused by DNA viral infection [45] (Figure 1). HPV upregulates UCHL1 to clear K63-linked ubiquitin chains from TRAF3, resulting in a lower amount of the downstream signaling complex TRAF3-TBK-1 to suppress the type I IFN pathway [46]. Further research is still needed to find and clarify the functions of DUBs during viral infection. More information will help control infectious diseases and facilitate the development of clinical antiviral therapies. 

## 4. DUBs Regulate Oncovirus Infection and Activation in an NF-*κ*B-Dependent Manner

RLR-, TLR-, and STING-induced innate immune response contribute to activation of NF-*κ*B. NF-*κ*B signaling plays an essential role in immune regulation and its role has been explored in almost all aspects of cellular activity. To achieve successful infection, oncoviruses have developed mechanisms to hijack the NF-*κ*B pathway. Multiple DUBs are key regulators of NF-*κ*B signaling. Several DUBs, such as CYLD and A20, have been extensively studied in the negative regulation of NF-*κ*B signaling. During the viral infection stage, HCV stimulation upregulates A20/ABIN1 expression, thereby suppressing NF-*κ*B activity and leading to inefficient M1 macrophage polarization to promote HCV infection [58]. EBV deubiquitinating Enzyme (v-DUB) BPLF1 inhibits TLR signaling through both MyD88- and TRIF-dependent pathways by removing ubiquitin chains from signaling intermediates, such as TRAF6, NEMO, and I*κ*B*α* [59, 60]. This leads to reduced NF-*κ*B activation and proinflammatory cytokine production in response to EBV and contributes to virus infectivity. During the infection stage, oncoviruses upregulate NF-*κ*B inhibitory DUBs or encode viral DUBs disrupting secretion of antiviral cytokines and interfering with the innate antiviral immune responses by inhibiting NF-*κ*B activation.

NF-*κ*B activation also plays an important role in virus reactivation, replication, and virus-mediated cell transformation. HIV inhibits CYLD to facilitate the NF-*κ*B pathway, playing an important role in HIV reactivation from latency [61]. HTLV-1- encoded Tax inactivates the NF-*κ*B negative regulators, A20 and CYLD, which allows chronic NF-*κ*B activation in HTLV-1-transformed cells [62]. USP20 deubiquitinates TRAF6 and Tax, thus suppressing interleukin 1*β* (IL-1*β*)- and Tax-induced NF-*κ*B activation, suggesting USP20 as a key negative regulator of Tax-induced NF-*κ*B signaling [63]. The HPV-encoded E6 protein targets CYLD, resulting in ubiquitination and proteasomal degradation of CYLD to induce NF-*κ*B activation [64]. In keratinocytes, HPV infection inhibits CYLD expression, resulting in enhanced K63-linked polyubiquitination and nuclear translocation of BCL-3, which leads to activation of the NF-*κ*B signaling pathway [65, 66]. Mutation of CYLD in HPV-positive head and neck squamous cell carcinomas (HNSCCs) leads to the activation of NF-*κ*B signaling and maintenance of episomal HPV in tumors. In KSHV-infected primary effusion lymphoma cell lines, KSHV-encoded viral FLICE inhibitory protein (vFLIP) K13 can induce NF-*κ*B activation, which upregulates A20 expression. A20 interacts with K13 and blocks K13-induced excessive NF-*κ*B activation in a negative feedback manner [67, 68]. The regulation of NF-*κ*B signaling by oncoviruses is not only important for the viral life cycle, but also contributes to the development of malignant tumors. Focusing on the role of DUBs in viral biology and NF-*κ*B may contribute to infection-related cancer prevention and treatment.

## 5. Oncoviruses Use Host DUBs or Encode v-DUBs to Facilitate Viral Infection and Replication

### 5.1. EBV

EBV-encoded latent membrane protein 1 (LMP1) is an important tumorigenic protein. Our previous studies have shown that LMP1 rescues p53-induced cell cycle arrest and apoptosis by promoting K63-linked ubiquitination of p53. LMP1 also inhibits cell necroptosis by modulating RIPK1/3(receptor interacting protein kinase 1/3) ubiquitination [69, 70]. LMP1 can also induce the expression of UCH-L1 and it may contribute to viral transformation and the progression of lymphoid malignancies [71, 72]. EBV nuclear antigen 1 (EBNA1) plays important roles in promoting EBV genome replication and persistence, and EBV latent gene expression. EBNA1 interacts with USP7, which is also known as herpes virus associated ubiquitin-specific protease (HAUSP). The EBNA1 and USP7 interaction can promote cell survival and contribute to EBNA1 functions at the EBV oriP and inhibit p53-mediated antiviral responses [73]. The EBV nuclear antigen 3 (EBNA3) family targets and interacts with USP46/USP12 deubiquitination complexes. The complex exhibits DUB activity and contributes to EBNA3-mediated lymphoblastoid cell growth [74]. Besides utilizing host DUBs, EBV can also encode the viral deubiquitinating enzyme, BPLF1, which is an immune evasion gene product that can suppress antiviral immune responses during primary infection [47]. BPLF1 is expressed during the late phase of lytic EBV infection and is incorporated into viral particles. It can eliminate K63- and/or K48-linked ubiquitin chains and act as an active DUB during the productive lytic cycle and EBV infection [48] (Table 3).

### 5.2. KSHV

KSHV-encoded viral interferon regulatory factor 1 (vIRF1) can bind to USP7 and decrease the deubiquitinase activity of USP7 for stabilizing p53, thereby disrupting the p53 signaling pathway [73]. Latency-associated nuclear antigen (LANA) induces the expression of UCH-L1, which might lead to viral transformation and the progression of lymphoid malignancies [71]. KSHV encoded tegument protein ORF64, which has deubiquitinase activity can inhibit the ubiquitination of RIG-I and suppress RIG-I-mediated IFN signaling. It is necessary for KSHV infection [49] (Table 3).

### 5.3. HPV

E6 and E7 are the main oncoproteins encoded by HPV. USP11 and USP15 can greatly increase the steady state level of HPV-16 E6 and E7 by reducing their ubiquitination and degradation, thereby increasing the oncogenic potential of HPV [75, 76].

### 5.4. HIV

HIV-1 Tat is encoded at an early stage after infection and is in charge of enhancing viral production. USP7 and USP47 stabilize the HIV-1 Tat protein by removing its K48 polyubiquitination chain [77]. The stabilization of Tat leads to enhanced* HIV-1* gene expression, facilitates virus spread, and also reduces immune recognition in HIV-1- expressing cells [78].

### 5.5. HCV

HCV encodes the core protein and nonstructural (NS) proteins NS3 and NS5A and promotes oncogenic transformation, replication, and virus assembly [9]. Studies show that NS5A binds to the ovarian tumor protein, deubiquitinase 7B (OTUD7B) and enhances OTUD7B DUB activity, which may contribute to viral replication and infection [50].

Oncoviruses utilize host DUBs to stabilize viral proteins, which increases the oncogenic potential of oncoviruses. Oncogenic viral products disturb host cell signaling pathways by enhancing the level of specific DUBs or DUB activity to promote viral genome replication and persistence. One DUB exhibited an opposite role in different oncoviruses, which indicates that if a DUB is used as an antiviral target, the potential effect on other viruses must be considered. Further studies are still needed to describe the detailed mechanisms between DUBs and oncoviruses.

## 6. DUB Inhibitors (DIs) as Potential Therapeutic Strategies

Inhibition of proteasome deubiquitinating activity is a new cancer therapy. Most DIs are small molecule compounds, exerting their function by suppressing DUB activity. The ubiquitin-specific proteases (USPs) are the largest and the most diverse DUB family and gene mutations, altered activity, or abnormal expression of USPs has been linked to multiple cancer types. USPs attractive are therapeutic targets and interest is growing in the development of enzyme selective or specific chemical inhibitors as antiviral and anticancer agents. The USP7-specific small molecule inhibitors, HBX41, 108, and P5091, induce apoptosis by stabilizing p53 in multiple myeloma cells resistant to conventional bortezomib therapies [55]. b-AP15 inhibits USP14 and UCHL5 and was shown to inhibit tumor growth in multiple solid tumor mouse models and attenuated tumor invasion in acute myelogenous leukemia in* in vivo *models [56]. WPI130 targets USP5, USP9X, and USP14 and inhibits viral progeny production of several RNA viruses, induces apoptosis, and suppresses growth of breast cancer cells [53, 57]. The USP1 inhibitors, GW7647 and ML323, attenuate growth of leukemic cells, non-small-cell lung cancer cells, and osteosarcoma cells [52, 54]. In light of these findings, DIs could be significant as potential therapeutic modalities in the treatment of multiple cancers. Given the multiple functions of DUBs in viral infection, developing inhibitors targeting the functional activities of virus-associated DUBs or virus-encoded DUBs might contribute to the reduction of oncovirus infections and could be used in infection-related cancers as accessory treatments (Table 4).

## 7. Conclusions and Perspectives

DUBs are central component in the ubiquitin signaling system to modulate proteostasis and have been shown to participate in all aspects of the viral life cycle. To escape from host immune responses, hijacking of the ubiquitin system by viruses continues to emerge as a central theme around virus infection and replication. In this review, we summarized recent studies focusing on the role of deubiquitinases in antiviral immune responses, modulation of the NF-*κ*B pathway, as well as on RNA and DNA oncovirus infection, replication, and pathogenesis. However, the detailed mechanisms between viruses, host, and DUBs are still not clear. As for the potential use of DIs as therapeutic strategies against cancer, many have been identified but none have been used clinically. As a new cancer therapy target, many challenges remain to be addressed for further understanding of DUBs function in order to develop compounds that inhibit or induce their activity to control the pathogenesis of oncoviruses.

## Figures and Tables

**Table 1:** Viral caused cancer types.

Virus type	Cancer-related virus	Cancer types	Mechanisms	Ref.
RNA virus	HIV-1	Lymphomas (most EBV-positive), KSHV-caused Kaposi sarcoma, and HPV-associated cervical and Anogenital carcinomas	indirect	[12]
	HTLV-1	Adult T-cell leukemia/lymphoma (ATL)	direct	[6, 11]
	HCV	Hepatocellular cancer, Non-Hodgkin lymphoma (especially B-cell lymphoma)	indirect	[14]
DNA virus	HBV	Hepatocellular cancer	indirect	[14]
	HPV	Cervix, Anal, Vulvar, and Penile cancers, and a subset of head and neck squamous cell carcinomas	direct	[1]
	KSHV	Kaposi sarcoma, primary effusion lymphoma	direct	[10]
	EBV	Nasopharyngeal carcinoma, Gastric cancer, Non-Hodgkin lymphomas (nhls), and Burkitt lymphoma, Nature killer/T-cell lyphoma	direct	[7–9]

**Table 2:** DUBs classification.

Categories	Families	DUBs
Cysteine proteases	USP	USP 1-8, USP 9X, USP 9Y, USP 10-16, USP 17 L1, USP 17 L2, USP 18-26, USP 27X, USP 28-54, USP L1, CYLD
	UCH	UCH L1, UCH L3, UCH L5, BAP1
	MJD	ATXN3, ATXN3L, JOSD1, JOSD2
	OTU	OTUB1, OTUB2, OTUD1, OTUD3, OTUD4, OTUD5, OTUD6A, OTUD6B, OTUD7A, OTUD7B, A2O, HIN1L, VCPIP1, TRABID, YOD1
	MINDY	FAM63A, FAM63B, FAM188A, FAM188B
Metalloproteases	JAMM	AMSH, AMSH-LP, BRCC36, COPS5, COPS6, EIF3F, EIF3H, MPND, MYSM1, PSMD7, PSMD14, PRPF8

Six classes of DUBs in the human genome are classified into two categories, cysteine proteases, and metalloproteases. Five classes are cysteine proteases: USP, ubiquitin-specific proteases; UCH, ubiquitin carboxyl-terminal hydrolases; MJD, Machado-Joseph disease protein domain proteases; OTU, ovarian-tumor proteases; MINDY, motif interacting with Ub-containing DUB family. One class is metalloproteases: JAMM, JAMM/MPN domain-associated metallopeptidases.

**Table 3:** Oncoviruses encoded v-DUBs.

oncovirus	v-DUB	Deubiquitination types	targets	pathways	Ref.
EBV	BPLF1	Lys48- or Lys63-linked polyubiquitin	TRAF6, NEMO, I*κ*B*α*	Inhibits TLR signaling and NF-*κ*B pathway	[47, 48]
KSHV	ORF64	Lys48- or Lys63-linked polyubiquitin	RIG-I	Inhibits RIG-I-mediated-IFN signaling	[49]

**Table 4:** Chemical DUB inhibitors.

DUB Inhibitors(DIs)	target	Cancer types	reference
HBX 41,108	USP5, 7, 8 and UCH-L3	myeloma	[50]
HBX -19,818	USP7	colon carcinoma	[51]
HBX-28,258	USP7	colon carcinoma	[51]
P5091	USP7	myeloma	[50]
P22077	USP7	-	[52]
GW7674	USP1	non-small cell lung cancer	[53, 54]
ML323	USP1 and some DUBs	non-small cell lung cancer and osteosarcoma	[53, 54]
b-AP15 (VLX1500)	UCHL5, USP14 and some DUBs	nonspecific	[55]
WPI 130	USP5/USP9x/USP14/UCHL1/UCHL5	breast cancer	[56, 57]
PR-619	broad-range DUB inhibitor	-	[52]
